# Burnout in healthcare education programs and English as a Medium of Instruction: the dark side of the language of instruction

**DOI:** 10.3389/fmed.2025.1619177

**Published:** 2025-07-30

**Authors:** Munassir Alhamami

**Affiliations:** Department of English, Faculty of Languages and Translation, King Khalid University, Abha, Saudi Arabia

**Keywords:** burnout, English as a Medium of Instruction (EMI), healthcare education, academic stress, student engagement, Saudi Arabia, language barriers

## Abstract

**Background:**

Current research findings show that students in healthcare education programs face high levels of burnout. When English is used as the Medium of Instruction (EMI), non-native speakers experience added linguistic strain, increasing their stress, cognitive overload, and emotional exhaustion.

**Purpose:**

This study explores the relationship between EMI and student burnout in undergraduate healthcare education programs in Saudi universities. It examines how EMI affects students’ emotional well-being, academic engagement, and sense of efficacy.

**Methods:**

A mixed-methods approach was used. Quantitative data were collected from 213 students using a burnout scale measuring four constructs: exhaustion, cynicism, academic efficacy, and disengagement. Qualitative data were gathered from open-ended responses, with thematic analysis guided by Maslach’s burnout framework.

**Results:**

Quantitative results showed high levels of exhaustion and disengagement, while cynicism and reduced efficacy were at moderate levels. Strong correlations were found between burnout dimensions, particularly between exhaustion, cynicism, and disengagement. Qualitative findings revealed that EMI caused emotional exhaustion due to linguistic overload, academic detachment from repeated failure, and reduced self-confidence in students’ academic skills. Many students expressed frustration, identity loss, and a desire to withdraw from their programs due to the use of EMI.

**Conclusion:**

The findings indicate that EMI can contribute significantly to student burnout in healthcare education. Without adequate language support and institutional adjustments, EMI may negatively affect students’ mental health, academic performance, and motivation. The study calls for more inclusive EMI practices that address students’ linguistic needs while protecting their well-being and academic success.

## Introduction

English as a Medium of Instruction (EMI) is expanding in Arab World universities, especially in Saudi Arabia (1, 2). EMI means teaching academic subjects in English in contexts where most students speak another first language (3). In healthcare education, EMI is often introduced to improve students’ global competitiveness. However, studying complex scientific subjects like medicine and pharamacy in English can be difficult for non-native speakers. It can lead to high levels of stress, mental fatigue, and cognitive overload (4).

These linguistic challenges become even more critical when placed within the already demanding structure of healthcare education. Students often face pressure from heavy workloads, clinical training, and high academic expectations. The use of EMI may add a further burden, especially when students do not receive enough language support. Recent research shows that burnout is common among healthcare students globally, with emotional exhaustion as a key symptom (5, 6). Burnout in academic settings includes three main components: emotional exhaustion, disengagement from studies, and reduced confidence in academic abilities (7).

In Saudi Arabia, these concerns are especially relevant. Most healthcare university undergraduate programs are taught in English, but many students start their university studies without strong English skills. This situation increases their risk of burnout. Language barriers can weaken students’ emotional well-being, academic engagement, and sense of efficacy.

This study investigates the connection between EMI and student burnout in undergraduate healthcare programs at Saudi universities. It uses a mixed-methods approach. The quantitative phase measures the levels of emotional exhaustion, cynicism, disengagement, and academic efficacy. The qualitative phase explores students’ personal experiences of learning healthcare subjects in English. The study aims to identify how EMI contributes to emotional exhaustion, academic challenges, and reduced motivation. It is important to note that this study focuses specifically on the emotional and psychological aspects of burnout in EMI contexts. While numerous studies have documented the benefits of EMI—such as increased global engagement and English proficiency—such advantages fall outside the scope of this research.

## Literature review

### Concept of burnout in academic settings

Burnout is a psychological condition caused by prolonged academic stress. It includes three key dimensions: emotional exhaustion, depersonalization, and reduced academic efficacy (7, 8). Emotional exhaustion means feeling mentally and physically drained. Depersonalization describes a negative or detached attitude toward studies. Reduced efficacy refers to a loss of confidence in one’s academic abilities.

Research shows that burnout is common among university students, especially in demanding fields like healthcare. Factors such as heavy workloads, weak social support, low optimism, and personal health issues increase the risk of burnout (9–11). As students advance through their programs, burnout symptoms often worsen, sometimes leading to dropout intentions (12, 13).

Burnout not only harms academic success but also affects emotional well-being. Students experiencing burnout often show lower academic performance, poor mental health, and a higher likelihood of quitting their studies (14–16). In healthcare education, these effects are particularly dangerous because emotional exhaustion can lower empathy, an important quality for clinical practice (17, 18). High levels of burnout have also been linked to professional misconduct and ethical failures among future healthcare professionals (19).

### Factors contributing to burnout in healthcare students

Several studies highlight the high prevalence of burnout among healthcare students. Almutairi et al. (5) found that 37% of medical students worldwide suffer from burnout, with emotional exhaustion being the most frequent symptom. Similarly, Frajerman et al. (6) reported a 44% burnout rate among medical students before entering residency programs. Different factors contribute to burnout in healthcare education. These include heavy academic pressure, fear of infection during the COVID-19 pandemic, and a lack of social and emotional support (20). Research among nursing students by Kong et al. (21) showed that 23% experienced burnout, often linked to intense workloads, sleep problems, and low self-confidence.

Institutional issues, such as rigid curricula and poor faculty support, also play a major role in increasing burnout risks (22). Furthermore, a lack of accessible mental health services worsens emotional exhaustion and reduces academic satisfaction (23). The impact of burnout extends beyond academic performance. Sinval et al. (24) showed that high burnout levels significantly increase students’ intentions to drop out. Factors like low psychological resilience, weak social support, and dissatisfaction with education are closely related to higher burnout. However, studies also reveal protective factors. Strong academic engagement, effective coping strategies, and supportive social environments help reduce burnout and its negative outcomes (25).

### The burden of EMI

In healthcare programs taught through EMI, the risk of burnout becomes even more severe. EMI places additional cognitive and emotional pressure on students, especially those who are non-native English speakers. Block (26) described this as the “dark side” of EMI, where language barriers increase academic stress and mental fatigue. Alhamami (27) highlighted that EMI often disadvantages students with limited English proficiency. These students face difficulties not only in understanding lectures but also in participating in class discussions and assessments. As a result, they experience higher levels of stress, frustration, and emotional exhaustion.

Alhamami (1) further confirmed that many healthcare students enrolled in EMI programs reported emotional exhaustion, low motivation, and difficulty understanding scientific lectures and specialized vocabulary. Gaffas (28) also found that Saudi students with lower English proficiency struggled significantly with reading, writing, speaking, and listening tasks. These challenges negatively affected both their academic performance and mental health. Alanazi and Curle (29) examined Saudi medical students and reported that many students struggled during their first years with academic writing, oral presentations, and lecture comprehension. This led to growing anxiety, reduced academic confidence, and a loss of interest in their field.

Similar patterns have been observed in international contexts. Yang et al. (30) studied Chinese medical students in EMI programs and found that unclear instruction, poor-quality teaching materials, and linguistic difficulties weakened students’ learning experiences. Even with supportive teaching methods, burnout remains a serious problem in EMI programs. For instance, Kushida and Troster (31) reported that over 80% of medical students enrolled in an EMI team-based learning program in Brazil suffered from burnout after three semesters. These findings across different countries suggest that EMI consistently increases students’ cognitive load and emotional strain, regardless of local educational practices or support systems.

To conclude, the literature clearly shows that burnout is widespread among healthcare students and that EMI adds an extra layer of cognitive and emotional strain. Language barriers in EMI programs lead to higher stress, reduced engagement, and lower academic performance. Studies consistently highlight the need for better institutional support, including English preparation programs, bilingual resources, flexible curricula, and mental health services. Addressing these issues is essential to protect students’ academic success, emotional health, and professional development. Universities implementing EMI must move beyond assuming that English proficiency is universal. Instead, they must design EMI policies and practices that respect and support the linguistic realities of their student populations.

This study makes an important contribution to research on EMI and burnout in healthcare education. It addresses a clear gap by focusing on how EMI affects students’ emotional well-being, academic engagement, and self-efficacy in Saudi universities. While previous studies explored burnout in medical education, few have examined the specific role of language barriers in causing emotional exhaustion and academic stress. The study fills a regional gap by focusing on the Arab world, where EMI is growing but often introduced without enough language support. Most research in this field has focused on academic outcomes rather than mental health. This study highlights the emotional and psychological struggles that students experience when studying complex subjects in English as non-native speakers.

It also addresses a methodological gap by using a mixed-methods design. By combining quantitative measures with thematic analysis of students’ written experiences, the study provides a richer and more complete understanding of how EMI contributes to burnout. The application of Maslach’s burnout framework to the EMI context addresses a theoretical gap. It shows how emotional exhaustion, disengagement, and reduced efficacy emerge in students facing both academic and linguistic challenges.

Practically, the study points to a mismatch between institutional EMI policies and students’ real needs. It shows that without proper preparation and bilingual support, EMI may harm students’ academic success and mental health. Finally, this study identifies new conceptual categories, such as linguistic overload, emotional collapse, and identity conflict. These offer a more detailed understanding of how burnout develops among EMI students. The findings suggest that more inclusive language policies are needed to support students’ academic and emotional well-being. The study is guided by three research questions:

Q1. What are the levels of emotional exhaustion, cynicism, academic efficacy, and disengagement among undergraduate healthcare students enrolled in EMI programs in Saudi universities?

Q2. How does EMI contribute to students’ emotional exhaustion, disengagement, and reduced academic efficacy in healthcare education programs?

Q3. What specific linguistic and academic challenges do students perceive as leading to psychological strain, reduced motivation, and withdrawal intentions in EMI healthcare education programs?

## Methodology

### Research design

This study employed a descriptive, cross-sectional research design within a mixed-methods framework to explore burnout among undergraduate students enrolled in EMI healthcare education programs at Saudi universities. The descriptive nature of the study allowed for the examination of current burnout levels and related experiences without manipulating variables. The cross-sectional approach captured data at a single point in time. The integration of both quantitative and qualitative data enabled a comprehensive analysis of students’ experiences. The quantitative phase measured the levels and interrelationships of burnout dimensions, while the qualitative phase offered deeper insights into the contextual and emotional aspects underlying students’ perceptions.

### Instruments

The quantitative instrument used in this study was a 17-item burnout scale that had been previously developed and validated in an EMI context across various university majors. The validation process involved around 500 students from different colleges and departments (Authors, under review). The scale measures four core constructs based on Maslach’s (8) framework: Exhaustion, Cynicism, Academic Efficacy, and Disengagement. Items were adapted from the Maslach Burnout Inventory–General Survey for Students (MBI-GS(S)) and the Oldenburg Burnout Inventory (OLBI), with contextual modifications to fit the EMI learning environment. As the instrument had already undergone comprehensive validation using Partial Least Squares Structural Equation Modeling (PLS-SEM) with a Reflective-Reflective Higher-Order Construct model, this study focused on applying the tool within healthcare and science programs without reassessing its psychometric properties. Responses were collected on a six-point Likert scale ranging from 1 (Strongly Disagree) to 6 (Strongly Agree), with reverse coding applied to negatively worded items to maintain consistent interpretation of scores. The six-point scale was deliberately chosen to exclude a neutral midpoint, which research has shown can introduce interpretive ambiguity and reduce response clarity. Midpoints are often interpreted inconsistently by respondents—as neutrality, indecision, or lack of engagement—potentially distorting findings in studies of psychological states like burnout (32, 33). By removing the midpoint, the scale prompts more decisive and interpretable responses. The survey included three sections: (a) Demographic Information (gender, university, college, year of study), (b) the Burnout Scale, and (c) two open-ended questions designed to capture participants’ reflections on the psychological and academic effects of EMI.

### Participants and sampling

A total of 213 undergraduate students participated in the study. Participants were drawn from 27 universities across Saudi Arabia, representing a diverse range of institutional contexts. Of the total sample, 154 students (72.3%) were enrolled in healthcare colleges (medicine, pharmacy, dentistry, applied medical sciences, and nursing), while 59 students (27.7%) were enrolled in science departments (biology, chemistry, biochemistry, physics, and biostatistics). The sample included students from all academic years, with a majority (51.6%) in their first year after the preparatory program.

### Data collection procedures

Ethical approval for the study was obtained from the Institutional Review Board (IRB), and all participants provided informed consent prior to participation. To accommodate linguistic diversity and enhance clarity, the survey was provided in both English and Arabic. Bilingual presentation of items helped reduce potential misinterpretation and cognitive strain, especially given the linguistic challenges inherent in EMI programs. Data were collected using an online survey developed via Google Forms. The survey link was distributed through multiple channels, including official university mailing lists, student WhatsApp groups, and Telegram platforms, which are widely used among Saudi university students. This multimodal distribution strategy enabled broader reach and participation across geographically and institutionally diverse student populations.

### Data analysis

#### Quantitative data

Descriptive and inferential analyses were conducted using Jamovi software. Burnout levels were categorized using a three-tier classification based on the six-point Likert scale, where 1.00–2.66 indicated low burnout, 2.67–4.33 indicated medium burnout, and 4.34–6.00 indicated high burnout. This categorization was derived by dividing the total scale range (1–6) into three equal intervals of approximately 1.66 points, a common approach in studies lacking established cut-offs. Pearson correlation coefficients were calculated to examine the relationships among the four burnout constructs. As the burnout scale had already been validated in a previous study conducted by the author (35), the present analysis focused solely on exploring burnout patterns in the sample without re-evaluating its psychometric properties.

#### Qualitative data

Open-ended responses were analyzed using thematic analysis. A total of 82 participants answered the first qualitative question and 72 answered the second. Most responses (over 90%) were written in Arabic and translated into English when needed. The analysis was guided by Maslach and Jackson’s (8) burnout framework, with codes grouped into three main categories: Emotional Exhaustion, Depersonalization (Disengagement), and Reduced Efficacy.

The researchers first read all responses carefully to understand the students’ experiences. They assigned short labels (codes) to important ideas. Related codes were then grouped into broader themes that directly emerged from the data. No pre-set categories were used. This approach allowed the analysis to reflect the students’ real experiences in EMI healthcare programs. Special care was taken to preserve the original meaning during translation. Words and phrases showing stress, fatigue, or loss of motivation were given particular attention. This helped the researchers identify how EMI influenced students’ emotional well-being and academic engagement. The final themes provided deeper insight into the emotional and academic challenges faced by students, complementing the quantitative findings of the study. As this is a single-author study, inter-coder reliability could not be conducted. However, rigorous procedures were followed to ensure trustworthiness, including multiple close readings, systematic coding, and iterative theme development.

## Results

### Descriptive statistics of participants

A total of 213 undergraduate students participated in the study. Among them, 154 (72.3%) were female and 59 (27.7%) were male. Regarding academic discipline, 145 students (68.1%) were enrolled in healthcare colleges, including programs such as medicine, pharmacy, dentistry, nursing, and applied medical sciences. The remaining 68 students (31.9%) were enrolled in science colleges, including departments such as biology, chemistry, biochemistry, and related fields.

Students came from 27 universities across Saudi Arabia. The largest groups were from Qassim University (12.2%), King Khalid University (11.3%), and Najran University (11.3%). Other universities, such as Majmaah University (9.4%) and Imam Mohammad Ibn Saud Islamic University (7.0%), contributed smaller proportions. Some universities, including King Saud University, Princess Nourah Bint Abdulrahman University, and Taif University, each contributed 4.7%. Institutions such as Batterjee Medical College, University of Bisha, and University of Hafr Al Batin were represented by one participant each.

Most students (51.6%) were in their first year of healthcare education. Second-year students made up 17.8%, third-year students 11.7%, fourth-year students 8.0%, and fifth-year students 7.5%. A small group (3.3%) had been enrolled for more than 5 years.

### Burnout levels

The burnout constructs were measured using a six-point Likert scale. Classification was based on dividing the scale into three equal intervals: low (1.00–2.66), medium (2.67–4.33), and high (4.34–6.00).

Table 1 shows that Exhaustion was classified as high. Cynicism and Academic Efficacy were classified as medium. Disengagement was classified as medium, close to the high threshold.

**Table 1:** Burnout results (*n* = 0.213).

Construct	Mean	SD	Range	Min	Max	Classification
Exhaustion	4.38	1.72	5	1	6	High
Cynicism	3.87	1.84	5	1	6	Medium
Academic Efficacy	3.23	1.63	5	1	6	Medium (note: reverse-coded)
Disengagement	4.26	1.93	5	1	6	Medium (near high)

### Correlation between burnout constructs

Pearson correlation coefficients were calculated among Exhaustion, Cynicism, Academic Efficacy, and Disengagement. Exhaustion and Cynicism showed a strong positive correlation (r = 0.864, *p* < 0.001). Exhaustion and Disengagement were strongly positively correlated (r = 0.871, *p* < 0.001). Cynicism and Disengagement also showed a strong positive correlation (r = 0.852, *p* < 0.001).

Academic Efficacy was negatively correlated with the other constructs: Exhaustion (r = −0.554, *p* < 0.001), Cynicism (r = −0.513, *p* < 0.001), Disengagement (r = −0.496, *p* < 0.001).

**Figure 1:**
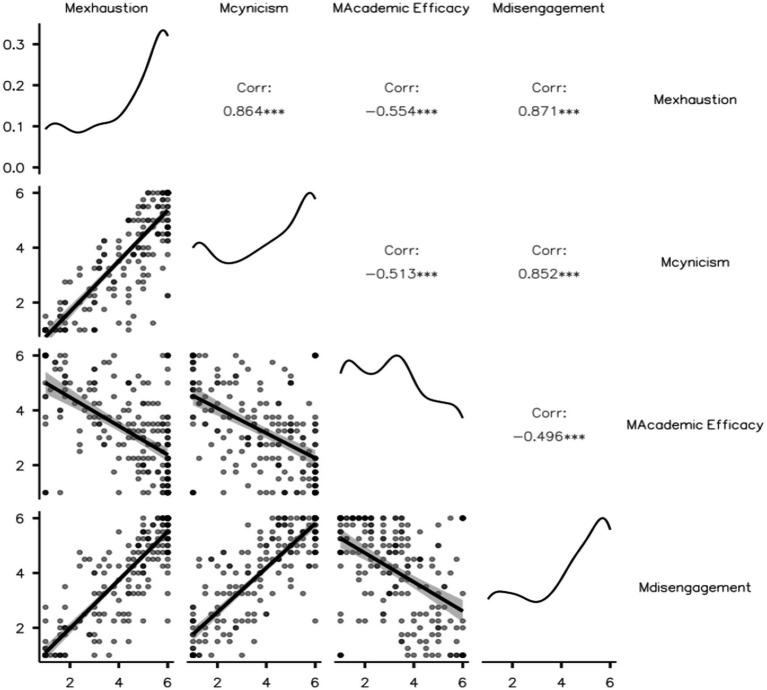
Scatterplot matrix of the four burnout constructs: exhaustion, cynicism, academic efficacy, and disengagement.

Figure 1 displays the scatterplot matrix of these relationships.

### Qualitative results

The qualitative component of this study explored students’ experiences with English as the language of instruction. Participants were asked two main questions: (1) how the use of English influenced their levels of psychological and mental exhaustion and emotional engagement with their studies, and (2) how English as the Medium of Instruction affected their academic efficacy and perceptions of the value of their major. Of the 213 participants, 82 provided complete responses to the first question and 72 provided complete responses to the second. Most responses were written in Arabic, with a small number in English. Incomplete or irrelevant answers were excluded. The analysis below presents the main themes identified from the participants’ comments.

Table 2 illustrates the categorization of themes related to Emotional Exhaustion, Depersonalization, and Reduced Efficacy in the context of EMI programs. The nine identified themes in this study were systematically grouped into three overarching categories that align with the well-established theoretical framework of burnout: emotional exhaustion, depersonalization, and reduced efficacy. These constructs provide a comprehensive lens through which to examine how EMI contributes to the psychological strain and academic disengagement experienced by healthcare students in non-native language contexts. These three dimensions do not operate in isolation. Emotional exhaustion typically arises first, as students attempt to navigate unfamiliar academic terrain in a foreign language. Over time, the persistent stress leads to emotional disengagement (depersonalization), followed by a diminished belief in one’s ability to succeed (reduced efficacy). Together, they form a self-perpetuating cycle of burnout.

**Table 2:** Major categories and themes of burnout experiences among EMI students.

Category	Theme
Emotional exhaustion	Cognitive Exhaustion
Emotional exhaustion	Emotional Fatigue and Psychological Collapse
Emotional exhaustion	Linguistic Burden
Emotional exhaustion	Burden of Translation and Rote Learning
Depersonalization and disengagement	Loss of Motivation and Passion for Learning
Depersonalization and disengagement	Disengagement and Withdrawal Intentions
Reduced efficacy and identity loss	Academic Alienation and Decline in Self-Efficacy
Reduced efficacy and identity loss	Identity Conflict and Behavioral Withdrawal
Reduced efficacy and identity loss	Institutional and Curricular Mismatch

#### Emotional exhaustion

Emotional exhaustion refers to the ongoing depletion of emotional and physical energy resulting from sustained academic and psychological demands. It is considered the most immediate dimension of burnout. In the present study, this construct emerged prominently among healthcare students enrolled in EMI programs. Participants reported significant strain associated with processing technical medical content in a non-native language. This included constant translation, decoding of unfamiliar terminology, and sustained cognitive effort, often accompanied by physical symptoms such as fatigue and headaches. Reported experiences highlighted a perceived mismatch between academic effort and achievement, contributing to continuous psychological strain. Emotional exhaustion was identified as a core entry point in the broader burnout process, leading to subsequent symptoms of disengagement and reduced academic efficacy.

##### Cognitive exhaustion

In Table 3, participants reported that studying in EMI imposed a significant cognitive burden, particularly when dealing with dense and technical content. Several participants described the general complexity of medical study in English (1.1.1), with others noting the challenge of specific courses like biology when taught in a second language (1.1.2). A recurring pattern was the dual task of understanding both the language and the scientific content, which was consistently described as mentally exhausting (1.1.3). Translation during lectures was identified as a major source of cognitive load, often consuming large amounts of time and energy (1.1.4). Despite repeated efforts, some students reported an ongoing inability to comprehend the material (1.1.5), while others expressed the mental intensity of the process in vivid terms (1.1.6). Together, these comments indicate that linguistic overload is a key contributor to emotional and cognitive exhaustion in EMI contexts.

**Table 3:** Examples of participants’ comments on cognitive exhaustion.

#	Arabic quote	English translation
1.1.1	دراسة sالطب باللغة الإنجليزية متعبة ومعقدة إلى حد ما.	Studying medicine in English is exhausting and somewhat complex.
1.1.2	دراسة مادة الأحياء البشري وما شابهها باللغة الإنجليزية أمر متعب جداً.	Studying human biology and similar subjects in English is very exhausting.
1.1.3	بذل مجهود ذهني هائل في محاولة فهم المحتوى … إنجاز مهمتين معاً، فهم اللغة وفهم المحتوى العلمي.	Exerting enormous mental effort trying to understand the content… doing two tasks at once, understanding the language and the scientific content.
1.1.4	أحتاج أترجم أغلب الكلام أو الشرح بالمحاضرات ويستهلك مني طاقة ووقت وجهد.	Translating most lectures drains my energy, time, and effort.
1.1.5	ما عاد صرت أفهم وحاولت بكل الطرق الفهم لكن لم أستطع أبداً.	I no longer understand; I’ve tried everything but failed.
1.1.6	أعصر مخي في عشرين مرة.	I squeeze my brain 20 times over.

##### Emotional fatigue and psychological collapse

In Table 4, participants frequently described early signs of academic exhaustion during their studies in English-medium programs. Several participants mentioned feeling study fatigue due to general exhaustion (1.2.1), with some indicating that this fatigue began very early in their academic experience (1.2.2). Physical symptoms such as headaches during lectures delivered in English were also reported (1.2.3). In more severe cases, participants described reaching critical levels of stress, with one noting a near loss of consciousness (1.2.4).

**Table 4:** Examples of participants’ comments on emotional fatigue and psychological collapse.

#	Arabic quote	English translation
1.2.1	الكثير من المواقف التي حسيت فيها بالإرهاق الدراسي بسبب الإجهاد.	Many situations where I felt study fatigue due to exhaustion.
1.2.2	الشعور بالإرهاق في فترة مبكرة.	Feeling exhausted at an early stage.
1.2.3	أشعر بالصداع عندما تُستخدم اللغة الإنجليزية في المحاضرة.	I get headaches when English is used in the lecture.
1.2.4	أكاد أن أجن وأفقد الوعي بسبب الضغط.	I’m on the verge of losing my mind and consciousness due to stress.
1.2.5	وأحس أني ضائعة.	I feel lost.
1.2.6	يعزز الشعور بالفشل.	It reinforces my sense of failure.
1.2.7	أحس باكتئاب.	I feel depressed.
1.2.8	مرهق.	Exhausted.

In addition to physical symptoms, students experienced strong emotional responses. Feelings of being lost were commonly expressed (1.2.5), alongside statements about reinforced feelings of failure (1.2.6). Some participants also reported symptoms of depression (1.2.7). Emotional exhaustion was summarized by others in simple but powerful terms, such as “Exhausted” (1.2.8). These comments show that emotional fatigue and psychological collapse were major challenges for students studying healthcare subjects in an EMI context.

##### Linguistic burden

In Table 5, participants consistently identified the language barrier as a major difficulty in their academic experience. Even basic subjects were described as requiring excessive effort when taught in English (1.3.1). The need to translate material frequently disrupted learning and extended study time (1.3.2). Participants noted that understanding concepts in Arabic did not translate into success when dealing with English-language assessments (1.3.3). The complexity of studying medicine in English was emphasized, with participants highlighting the exhausting and confusing nature of the language load (1.3.4). Language-specific difficulties in exams often led to incorrect answers, even when students understood the underlying concepts (1.3.5). Some described particular challenges in studying non-technical subjects like health ethics when the material was presented only in English (1.3.6). These quotes show that linguistic burden was a persistent obstacle to academic achievement in EMI healthcare programs.

**Table 5:** Examples of participants’ comments on linguistic burden.

#	Arabic quote	English translation
1.3.1	حتى أبسط المواد تأخذ مجهودًا كبيرًا، لو كانت بلغة أفهمها لما أخذت هذا الجهد والتعب.	Even the simplest subjects take great effort; if they were in a language I understand, they would not take all this effort and fatigue.
1.3.2	صعوبات في الدراسة والتي قد تمتد إلى ساعات بسبب الترجمة.	Study difficulties that may extend to hours due to translation.
1.3.3	فاهمة الشيء بالعربي بس لما أشوف الأسئلة بالإنجليزي كأنني أول مرة أشوفها.	I understand the concept in Arabic, but when I see the questions in English, it’s as if I’m seeing it for the first time.
1.3.4	دراسة الطب باللغة الإنجليزية متعبة ومعقدة إلى حد ما.	Studying medicine in English is exhausting and somewhat complex.
1.3.5	يكون فيه مصطلح في السؤال مما يجعل حلي خاطئ.	A term in the question makes my answer wrong.
1.3.6	أواجه اختبار أخلاقيات المهن الصحية وكله بالإنجليزي ويصعب مذاكرته جدًا.	Health ethics exams in English are extremely hard to study.

##### Burden of translation and rote learning

In Table 6, participants reported that translation tasks extended their study hours, often taking up entire evenings (1.4.1). Many noted feeling mentally drained from translating lectures before they could begin actual studying (1.4.2). Some had to revisit the same topic in multiple languages to grasp basic concepts (1.4.3). Translation activities dominated their schedules, reducing time available for deeper academic engagement (1.4.4). The pressure to translate rapidly, despite time constraints, contributed to inefficient and stressful learning experiences (1.4.5). Participants often required more time than usual to complete their academic tasks due to the burden of translation and rote memorization (1.4.6). These experiences highlight how translation demands acted as a major barrier to effective learning in EMI contexts.

**Table 6:** Examples of participants’ comments on burden of translation and rote learning.

#	Arabic quote	English translation
1.4.1	أخذ ساعات طويلة لتتم عملية الترجمة.	It takes many hours to complete the translation process.
1.4.2	أحتاج أترجم أغلب الكلام أو الشرح بالمحاضرات ويستهلك مني طاقة ووقت وجهد.	I need to translate most of the lecture content, and it consumes my energy, time, and effort.
1.4.3	أدرس المادة مرة فهم بالعربي وخمس مرات بالإنجليزي.	I study the subject once in Arabic and five times in English.
1.4.4	الترجمة تأخذ أغلب وقتك.	Translation consumes most of your time.
1.4.5	نرجع ومافي وقت بس لازم نترجم المحاضرة عشان نفهم.	We return with no time but must translate lectures to understand.
1.4.6	أحتاج وقتًا أكثر من اللازم لدراسته.	I need excessive time to study.

#### Disengagement and depersonalization

Disengagement and depersonalization refer to the emotional withdrawal and psychological distancing that individuals develop in response to sustained academic stress. In this study, these symptoms emerged prominently among participants enrolled in EMI programs. Many participants reported a gradual loss of academic motivation and a fading connection to their healthcare studies. Feelings of disillusionment with their majors and the academic environment were common. Disengagement often followed repeated academic struggles despite continuous effort, particularly when participants perceived a gap between their efforts and academic outcomes. This detachment functioned as a coping response to ongoing frustration and emotional fatigue. Participants showed reduced emotional investment in their studies, lower participation in academic activities, and a growing sense of alienation from their educational goals. Disengagement and depersonalization appeared as critical stages in the progression of burnout, reinforcing emotional exhaustion and leading to diminished academic persistence.

##### Loss of motivation and passion for learning

In Table 7, participants reported that studying in EMI programs led to a gradual loss of motivation and passion for their academic fields. Some participants described how their feelings toward their major changed negatively over time (2.1.1). The shift from studying in Arabic to studying in English reduced their enthusiasm for learning (2.1.2). Academic engagement became mechanical, focused only on passing exams rather than genuine understanding or curiosity (2.1.3). Several participants reflected on a perceived decline in their academic performance and identity compared to their high school achievements (2.1.4). They expressed frustration at feeling left behind during lectures, regardless of their continued efforts (2.1.5). These experiences indicate that language barriers and constant academic pressure contributed significantly to the erosion of motivation and passion among students in EMI healthcare programs.

**Table 7:** Examples of participants’ comments on loss of motivation and passion for learning.

#	Arabic quote	English translation
2.1.1	حالياً كرهت تخصصي.	Right now I hate my major.
2.1.2	فقدت حماسي السابق عندما كنت أدرس أي مادة بالعربية.	I lost the enthusiasm I once had when I used to study any subject in Arabic.
2.1.3	من أجل الاختبار فقط.	Just for the exam.
2.1.4	اللي كنا متفوقين أيام الثانوي ودائماً مشاركين صرنا الأسوأ بالجامعة.	Those of us who were top participants in high school and always participated have become the worst at university.
2.1.5	في المحاضرات الدراسية أشعر بكثير من التأخر.	In lectures I feel very behind.

##### Disengagement and withdrawal intentions

In Table 8, participants reported frequent thoughts about withdrawing from their programs or transferring to majors taught in Arabic. These thoughts were serious reactions to ongoing emotional and academic pressure (2.2.1). Feelings of confinement and isolation were common, with participants describing the university experience as restrictive and overwhelming (2.2.2). Some participants linked their loss of passion directly to the challenges of studying in English (2.2.3). A mismatch between personal expectations and the university environment was also frequently mentioned (2.2.4). In some cases, participants expressed urgent desires to quit their studies completely due to the accumulated stress (2.2.5). These comments indicate that language barriers and persistent academic strain in EMI programs contributed significantly to disengagement and withdrawal intentions among students.

**Table 8:** Examples of participants’ comments on disengagement and withdrawal intentions.

#	Arabic quote	English translation
2.2.1	أفكار مثل الإنسحاب من الجامعة أو التحويل لتخصص يُدرّس باللغة العربية.	Thoughts like withdrawing from the university or switching to a major taught in Arabic.
2.2.2	احس اني مسجون.	I feel imprisoned.
2.2.3	حالياً كرهت تخصصي.	Now I hate my major.
2.2.4	الجامعة مو مناسبة لي وان ذا مو مكاني.	The university is not suitable for me; this is not my place.
2.2.5	وابى انسحب وما اكمل دراسة في هذا التخصص بأي طريقة.	I want to withdraw and quit this major by any means.

#### Reduced efficacy and identity loss

Reduced efficacy refers to a diminished sense of competence, achievement, and academic control, often arising in contexts of sustained academic challenge. In this study, reduced efficacy was a prominent experience among participants enrolled in EMI programs. Participants reported that their solid understanding of medical content in Arabic did not translate into academic success when assessed in English. Difficulties in navigating English-language assessments, particularly when unfamiliar terminology was involved, contributed to feelings of frustration and demoralization. Many participants perceived that exams assessed language ability more than subject mastery, further weakening their academic confidence. The absence of adequate institutional support, such as English placement tests and bilingual learning resources, intensified these challenges. Over time, the repeated mismatch between effort and academic achievement led participants to internalize feelings of inadequacy, self-doubt, and academic failure. Reduced efficacy, combined with identity loss, emerged as a critical dimension of the burnout process, deepening emotional exhaustion and disengagement.

##### Academic alienation and decline in self-efficacy

In Table 9, participants described significant challenges during exams and academic interactions. Many felt that exams tested English language skills rather than content knowledge (3.1.1). Difficult English terminology in exam questions caused even well-prepared participants to make mistakes (3.1.2). Language limitations also affected oral participation in class. Participants reported avoiding discussions and answers due to low English vocabulary and communication anxiety (3.1.3). This lack of confidence limited engagement and increased feelings of alienation from their academic environment. Several participants explained that they understood course material conceptually but struggled to perform well due to language barriers (3.1.4, 3.1.5). A repeated mismatch between effort and grades created a sense of helplessness and resignation (3.1.6). These comments illustrate how linguistic barriers not only impaired performance but also damaged participants’ academic confidence and motivation over time.

**Table 9:** Examples of participants’ comments on academic alienation and decline in self-efficacy.

#	Arabic quote	English translation
3.1.1	كان الاختبار يعتمد على اللغة اكثر من اعتماده على فهم الطالب.	The exam relied more on language than on the participant’s understanding.
3.1.2	يكون فيه مصطلح في السؤال مما يجعل حلي خاطئ.	Sometimes there’s a term in the question that makes my answer wrong.
3.1.3	لا يمكنني التفاعل والفهم والاجابة بسبب قله المصطلحات الانجليزية.	I cannot interact, understand, or answer because of my limited English vocabulary.
3.1.4	فأهمه المادة كامل بس.	I completely understand the material, but…
3.1.5	رغم ان لو فهمت معناة استطعت حله.	If I had understood its meaning, I could have solved it.
3.1.6	اقعد على السلايدة كثير لين شي يقول خلاص درجاتك نازلة حتى لو ذاكرتي فأرتاحي.	I spend a long time on the slides until something tells me, ‘Your grades are low anyway, even if you study, so just rest’.

##### Identity conflict and behavioral withdrawal

In Table 10, participants described a significant shift in their academic identity. Many transitioned from being high achievers in high school to feeling academically weak at the university level (3.2.1). Some attended classes only to meet attendance requirements, without meaningful engagement (3.2.2). Reduced achievement was commonly reported and linked to feelings of inadequacy and frustration (3.2.3). The emotional burden of EMI translated into physical symptoms, including frequent headaches during English lectures (3.2.4) and general physical exhaustion (3.2.5). Severe mental strain was also mentioned, with some participants approaching emotional collapse (3.2.6). Language barriers further contributed to participants’ academic and professional struggles. Many felt unable to interact or answer questions due to limited English vocabulary (3.2.7). Fear of embarrassment prevented open participation in classroom discussions (3.2.8). These experiences illustrate how linguistic challenges in EMI environments affect not only academic performance but also students’ psychological well-being and professional development.

**Table 10:** Examples of participants’ comments on identity conflict and behavioral withdrawal.

#	Arabic quote	English translation
3.2.1	الي كنا متفوقين ايام الثانوي و دائما مشاركين صرنا الأسوء بالجامعة.	We were top participants in high school but became the worst at university.
3.2.2	فقط احظر من اجل ان لاتزيد نسبة الغياب ويجيني حرمان.	I attend only to avoid being barred for absences.
3.2.3	وبالتالي قلة الإنجاز.	Hence, reduced achievement.
3.2.4	اشعر بالصداع عندما تُستخدم اللغة الإنجليزية في المحاضرة.	I get headaches when English is used in lectures.
3.2.5	تعب جسدي.	Physical fatigue.
3.2.6	اكاد ان اجن وافقد الوعي بسبب الضغط.	I’m on the verge of mental breakdown from pressure.
3.2.7	لا يمكنني التفاعل والفهم والاجابة بسبب قله المصطلحات الانجليزية.	I cannot interact, understand, or respond due to limited English terms.
3.2.8	الشعور بالخجل من التحدث أمام الزملاء.	Feeling ashamed to speak in front of peers.

##### Institutional and curricular mismatch

In Table 11, participants frequently expressed a strong preference for studying in Arabic, believing it would lead to faster and deeper learning (3.3.1, 3.3.2). Some participants attributed their difficulties to a late start in English education during primary school (3.3.3). Many emphasized that studying in their native language would not only reduce academic strain but also make learning more enjoyable and efficient (3.3.4, 3.3.5). Institutional shortcomings further complicated participants’ experiences. The absence of English placement tests upon university entry was a common concern (3.3.6). Communication difficulties with non-Arabic-speaking faculty members were frequently mentioned (3.3.7), along with complaints about insufficient English preparation during secondary education (3.3.8). Without adequate support, many participants turned to alternative strategies such as relying on AI tools or peer assistance to manage their coursework (3.3.9). These comments highlight the critical need for systematic language support, bilingual resources, and curricular reforms to better accommodate students in EMI healthcare programs.

**Table 11:** Examples of participants’ comments on institutional and curricular mismatch.

#	Arabic quote	English translation
3.3.1	ياليت لو كان بالعربي والله والله امنية.	I wish it were in Arabic, really, it’s a dream.
3.3.2	لماذا لا ندرس بلغتنا العربية؟	Why do not we study in our Arabic language?
3.3.3	من الابتدائي ما اخذنا انقلش الا سادس.	We did not start English until Grade 6.
3.3.4	عكس لما أدرسه بلغتي العربية بيكون سهل جدا وممتع.	Studying in Arabic is easier and enjoyable.
3.3.5	أقدر أفهم بلغتي العربية بشكل أسرع وأكثر دقة من اللغة الإنجليزية.	I understand faster and more accurately in Arabic.
3.3.6	الجامعة مافيها اختبار تحديد مستوى للانجليزي.	The university has no English placement test.
3.3.7	صعوبة في التواصل مع الدكاترة خصوصاً إذا لم يكونوا ناطقين باللغة العربية.	Difficulty communicating with non-Arabic-speaking professors.
3.3.8	عدم تطوير لغتنا في المرحلة الثانوية.	Our English wasn’t developed in high school.
3.3.9	يؤدي لاستخدام ذكاء اصطناعي وطلب المساعده.	[This] leads to using AI and asking for help.

## Discussion

This study explored the relationship between EMI and burnout among undergraduate healthcare participants in Saudi universities. Using a mixed-methods approach, the study revealed significant emotional, cognitive, and academic challenges associated with EMI, contributing to elevated levels of burnout.

Quantitative results indicated that emotional exhaustion reached a high level, while cynicism and disengagement were at moderate to near-high levels. Academic efficacy was moderately low. Strong positive correlations were observed among exhaustion, cynicism, and disengagement, while academic efficacy was negatively correlated with the other burnout constructs. These results suggest that EMI contributes significantly to emotional fatigue, disengagement, and reduced academic confidence among participants. These findings align with prior research identifying emotional exhaustion as a core feature of student burnout in healthcare education (5, 6).

The first major finding relates to emotional exhaustion, which appeared as the most common and intense experience. Participants reported constant mental and emotional strain due to the pressure of understanding complex scientific content in a foreign language. The effort required to translate lectures, memorize unfamiliar terms, and sustain attention during English-language instruction often led to physical symptoms such as headaches and persistent fatigue. Similar patterns of psychosomatic symptoms and mental fatigue have been reported in previous studies on EMI-induced stress (28, 30). A clear mismatch was identified between participants’ academic efforts and their perceived outcomes, reinforcing feelings of stress and helplessness. This emotional exhaustion marked the early stage of the burnout process, consistent with Maslach and Leiter’s (7) conceptualization of burnout progression.

The second major finding concerns disengagement and depersonalization. Continued emotional stress led participants to lose motivation and emotional connection to their healthcare studies. Many participants reported a loss of passion for their majors, describing their academic engagement as mechanical and exam-focused. Former high-achieving participants in Arabic-medium education shared that they felt alienated and academically unsuccessful in EMI environments, a phenomenon previously described in EMI literature (26, 27). Some participants considered withdrawing from their programs or transferring to Arabic-medium instruction, highlighting the depth of their emotional distancing. This psychological withdrawal served as a coping mechanism but further weakened academic persistence.

Reduced efficacy was the third major theme. Many participants felt that their understanding of medical content in Arabic did not translate into success when assessed in English. Exams were perceived as testing language ability more than scientific knowledge, a difficulty similarly observed by Alanazi and Curle (29). This perceived disconnect undermined participants’ academic identity and confidence. Feelings of academic helplessness were compounded by limited institutional support, such as the absence of English placement tests and lack of bilingual resources. Participants internalized these challenges, resulting in persistent self-doubt and a diminished belief in their academic abilities. These findings are aligned with previous studies noting the critical role of language preparedness in shaping EMI student outcomes (28, 30).

The qualitative findings expand the quantitative results by illustrating how linguistic overload, cognitive fatigue, and emotional collapse are deeply interconnected. Participants consistently reported that the dual demands of language decoding and content learning slowed their academic progress, reduced comprehension, and increased emotional exhaustion. Translation efforts dominated study routines, leading to inefficient learning and weakened mastery of healthcare subjects, echoing concerns raised by Kushida and Troster (31) about burnout in EMI environments.

Moreover, participants reported that EMI negatively impacted their broader well-being. Many described long study hours, lack of sleep, physical exhaustion, and social isolation. Some participants felt disconnected not only from academic communities but also from family and friends, further deepening emotional fatigue. Similar findings have been reported in EMI healthcare contexts, where emotional and social isolation were found to exacerbate academic stress (1, 26). Over time, the combination of linguistic barriers, academic failure, and physical stress contributed to severe burnout symptoms, including withdrawal intentions and loss of career motivation.

These findings align with previous research identifying a foreign language as the medium of instruction overload, stress, and disengagement in higher education settings (1, 26, 30). However, this study extends prior work by focusing specifically on healthcare education in Saudi Arabia and by highlighting the emotional, psychological, and academic consequences of EMI on non-native English-speaking participants. The study extends the concept of the “dark side” of EMI, as framed by Block (26), by demonstrating its psychological cost in a regionally specific context.

At the institutional level, the results highlight critical systemic gaps. The absence of structured English support, lack of bilingual scaffolding, and insufficient academic guidance exacerbated participants’ difficulties. Without targeted interventions, EMI policies risk creating academic inequities and increasing emotional strain among students who are not adequately prepared for English-based education (28, 29).

Practically, these findings suggest that universities implementing EMI in healthcare programs must offer more inclusive and supportive strategies. Establishing preparatory English programs, offering bilingual learning materials, training faculty in inclusive teaching methods, and providing mental health support services are necessary steps. Similar recommendations have been proposed in international EMI research aiming to reduce linguistic and academic barriers (1, 30).

Universities must also recognize that linguistic preparation is an essential factor for academic success in EMI settings, not a peripheral concern. Without comprehensive institutional support, the pressure to study complex healthcare content in English can lead to emotional exhaustion, disengagement, reduced academic efficacy, and potential dropout. Implementing reforms that address both language and academic support needs can help protect participants’ mental health and educational outcomes.

Recent global research has underscored the urgent need to reassess language policies in medical education, particularly in countries where foreign languages serve as the medium of instruction. A worldwide screening by Hamad (34) revealed that while most developed countries deliver medical education in the mother tongue, many developing countries—especially in Africa and the Arab world—continue to rely on colonial languages such as English and French. This reliance, driven more by historical and political factors than by pedagogical considerations, contributes to academic and communicative challenges for domestic students. Building on this, Hamad et al. (2) conducted a systematic review of 49 studies involving over 14,500 students and identified two major consequences of foreign-language-based medical education: diminished academic performance due to difficulty understanding foreign-language materials, and impaired patient communication during clinical training. The review emphasizes that students trained in their native language demonstrate greater confidence, empathy, and clarity in clinical interactions. Both studies advocate for policy reforms that prioritize native-language instruction and propose bilingual approaches or supplemental native-language modules as feasible solutions. These findings provide strong support for integrating institutional language support and localized pedagogy to mitigate burnout and cognitive overload in EMI healthcare education.

### Limitations and future research

This study has several limitations that should be considered. First, the sample was limited to undergraduate participants enrolled in healthcare-related programs such as medicine, pharmacy, dentistry, and applied sciences. The results may not generalize to participants from other academic disciplines, such as engineering, humanities, or business, where academic and linguistic challenges may differ. Future research should examine burnout experiences across a broader range of fields. Second, the study focused solely on undergraduate programs. It did not explore the experiences of participants in postgraduate or professional training programs, who may encounter different academic pressures and language demands. Future studies should investigate how EMI impacts burnout across different academic levels. Third, the study was conducted in Saudi Arabia, where most participants share the same first language, Arabic. This linguistic homogeneity may have influenced how participants experienced EMI and burnout. Researchers in multilingual or different cultural settings may observe distinct patterns. Comparative studies across diverse linguistic and national contexts are recommended.

Additionally, the study relied primarily on self-reported data collected through questionnaires and open-ended responses. This approach may introduce biases related to participants’ perceptions or emotional states at the time of data collection. Future research could incorporate academic performance records, interviews, and observational data to provide a more comprehensive and objective view. Moreover, as the study employed a cross-sectional design, it captures experiences at a single point in time. Longitudinal studies are needed to track the development of burnout symptoms throughout participants’ academic journeys, from entry to graduation, in EMI programs.

To strengthen future research, it is important to expand the focus beyond participants’ experiences alone. Including the perspectives of instructors, program directors, and policy-makers could offer a fuller understanding of how EMI practices, institutional support systems, and language policies impact burnout. Studies that compare EMI and Arabic-Medium Instruction (AMI) programs directly would also help clarify the specific contribution of language barriers to burnout outcomes. Furthermore, using in-depth interviews or focus groups could allow researchers to explore participants’ emotional and cognitive experiences in greater depth. Research should also examine the effectiveness of various interventions—such as preparatory language courses, bilingual teaching strategies, and academic counseling services—on reducing burnout in EMI healthcare education. Longitudinal designs, collecting data at multiple points across academic programs, would provide valuable insight into how burnout symptoms evolve over time and how participants adapt or fail to adapt to EMI pressures. Finally, it should be noted that this study does not attempt to evaluate the full range of EMI outcomes. Instead, it concentrates solely on the burnout-related challenges faced by students in EMI healthcare programs. A detailed comparison with positive EMI impacts is beyond the study’s scope and would detract from the central objective, which is to draw attention to the emotional and academic strain that EMI may impose in under-supported educational environments.

## Conclusion

This study demonstrates that EMI significantly contributes to burnout among undergraduate participants in healthcare education in Saudi Arabia. Emotional exhaustion, disengagement, and reduced efficacy were prominent experiences linked to linguistic overload, academic challenges, and lack of institutional support. Without appropriate reforms, EMI can intensify educational inequities and compromise participants’ emotional health and academic success. To mitigate these effects, universities should consider implementing mandatory pre-EMI language assessments to ensure student readiness, as well as bilingual teaching models that ease the cognitive load during instruction. Additional measures such as structured English language support, localized curricula, inclusive pedagogies, and integrated mental health services are essential. These interventions can reduce burnout and promote more equitable and effective EMI experiences in healthcare education.

## Data Availability

The raw data supporting the conclusions of this article will be made available by the authors, without undue reservation.

## Author’s note

MA is a full professor of applied linguistics whose research interests include language of instruction, learners’ beliefs, online learning, ESP, and mixed methods research.

## References

[1] Alhamami M. One decade of “English as a medium of instruction” (EMI) in healthcare education. Front Med. (2024) 11:1296563. doi: 10.3389/fmed.2024.1296563, PMID: 38487028 PMC10937345

[2] Hamad AA Mustaffa DB Alnajjar AZ Amro R Deameh MG Amin B. Decolonizing medical education: a systematic review of educational language barriers in countries using foreign languages for instruction. BMC Med Educ. (2025) 25:701. doi: 10.1186/s12909-025-07251-2, PMID: 40361088 PMC12077016

[3] Dearden J. English as medium of instruction: A growing global phenomenon. London: British Council (2014).

[4] Alhamami M Almelhi A. English or Arabic in healthcare education: perspectives of healthcare alumni, students, and instructors. J Multidiscip Healthc. (2021) 14:2537–47. doi: 10.2147/JMDH.S330579, PMID: 34552332 PMC8450159

[5] Almutairi H Alsubaiei A Abduljawad S Alshatti A Fekih-Romdhane F Husni M. Prevalence of burnout in medical students: a systematic review and meta-analysis. Int J Soc Psychiatry. (2022) 68:1157–70. doi: 10.1177/00207640221106691, PMID: 35775726

[6] Frajerman A Morvan Y Krebs MO Gorwood P Chaumette B. Burnout in medical students before residency: a systematic review and meta-analysis. Eur Psychiatry. (2019) 55:36–42. doi: 10.1016/j.eurpsy.2018.08.006, PMID: 30384110

[7] Maslach C Leiter MP. The burnout challenge: Managing people’s relationships with their jobs Harvard University Press (2022).

[8] Maslach C Jackson SE. The measurement of experienced burnout. J Organ Behav. (1981) 2:99–113. doi: 10.1002/job.4030020205

[9] Boni RADS Paiva CE de Oliveira MA Lucchetti G Fregnani JHTG Paiva BSR. Burnout among medical students during the first years of undergraduate school: prevalence and associated factors. PLoS One. (2018) 13:e0205987. doi: 10.1371/journal.pone.0205987 29513668 PMC5841647

[10] Jezzini-Martínez S Quiroga-Garza A Jacobo-Baca G Guzmán-López S Martínez-Garza JH. COVID-19 causing burnout among medical students. FASEB J. (2021) 35:7. doi: 10.1096/fasebj.2021.35.S1.04749

[11] Kilic R Nasello JA Melchior V Triffaux JM. Academic burnout among medical students: respective importance of risk and protective factors. Public Health. (2021) 198:187–95. doi: 10.1016/j.puhe.2021.07.025, PMID: 34478954

[12] Pagnin D de Queiroz V de Oliveira Filho MAE Gonzalez NVA Salgado AET Oliveira BC. Burnout and career choice motivation in medical students. Med Teach. (2013) 35:388–94. doi: 10.3109/0142159X.2013.769673 23458255

[13] Thun-Hohenstein L Höbinger-Ablasser C Geyerhofer S Lampert K Schreuer M Fritz C. Burnout in medical students. Neuropsychiatrie. (2020) 35:17–27. doi: 10.1007/s40211-020-00359-5, PMID: 32880881 PMC7954737

[14] Carmona-Halty M Alarcón-Castillo K Semir-González C Sepúlveda-Páez G Schaufeli WB. Burnout assessment tool for students (BAT-S): evidence of validity in a Chilean sample of undergraduate university students. Front Psychol. (2024) 15:1434412. doi: 10.3389/fpsyg.2024.1434412, PMID: 39744027 PMC11688192

[15] Madigan DJ Curran T. Does burnout affect academic achievement? A meta-analysis of over 100,000 students. Educ Psychol Rev. (2021) 33:387–405. doi: 10.1007/s10648-020-09533-1

[16] Schaufeli WB Martínez IM Pinto AM Salanova M Bakker AB. Burnout and engagement in university students: a cross-national study. J Cross-Cult Psychol. (2002) 33:464–81. doi: 10.1177/0022022102033005003

[17] von Harscher H Desmarais N Dollinger R Grossman S Aldana S. The impact of empathy on burnout in medical students: new findings. Psychol Health Med. (2018) 23:295–303. doi: 10.1080/13548506.2017.1374545 28954529

[18] Zakerkish M Askari S Nazari A. Association between burnout and empathy in medical residents. PLoS One. (2024) 19:e0283497. doi: 10.1371/journal.pone.0301636 PMC11003688 38593142

[19] McTaggart LS Wilson MC Bell KM. The relationship between resident physician burnout and its effects on patient care, professionalism, and academic achievement: a review of the literature. Health Sci Rev. (2022) 5:100059. doi: 10.1016/j.hsr.2022.100059

[20] Peng P Hao Y Liu Y Chen S Wang Y Yang Q. The prevalence and risk factors of mental problems in medical students during COVID-19 pandemic: a systematic review and meta-analysis. J Affect Disord. (2023) 321:167–81. doi: 10.1016/j.jad.2022.10.040, PMID: 36341802 PMC9613786

[21] Kong L-N Yao Y Chen S-Z Zhu J-L. Prevalence and associated factors of burnout among nursing students: a systematic review and meta-analysis. Nurse Educ Today. (2023) 121:105706. doi: 10.1016/j.nedt.2022.105706, PMID: 36577286

[22] Duke N Reiser J Robinson G. Institutional factors associated with burnout among assistant professors. Teach Learn Med. (2020) 32:482–91. doi: 10.1080/10401334.2019.1638263 31315454

[23] Sadikan MZ. Addressing burnout in medical students and residents: strategies for sustainable well-being. Int J Transform Health Profess Educ. (2024) 3:15–24. doi: 10.71354/ijthpe.02.01.23

[24] Sinval J Oliveira P Novais F Almeida CM Telles-Correia D. Correlates of burnout and dropout intentions in medical students: a cross-sectional study. J Affect Disord. (2024) 364:221–30. doi: 10.1016/j.jad.2024.08.003, PMID: 39128773

[25] Abreu Alves S Sinval J Lucas Neto L Marôco J Gonçalves Ferreira A Oliveira P. Burnout and dropout intention in medical students: the protective role of academic engagement. BMC Med Educ. (2022) 22:83. doi: 10.1186/s12909-021-03094-9, PMID: 35130892 PMC8821797

[26] Block D. The dark side of EMI? A telling case for questioning assumptions about EMI in higher education. Educ Linguist. (2022) 1:82–107. doi: 10.1515/eduling-2021-0007

[27] Alhamami M. Inequity, inequality, and language rights in English as a medium of instruction programs. Eval Program Plann. (2023) 99:102297. doi: 10.1016/j.evalprogplan.2023.102297, PMID: 37167792

[28] Gaffas ZM. Language challenges in medical education: exploring predictors and variations among EMI students in Saudi Arabia. AILA Review. Advance online publication. (2025). doi: 10.1075/aila.24013.gaf

[29] Alanazi K Curle S. Challenges experienced by students studying medicine through English medium instruction. Front Educ. (2024) 9:1364860. doi: 10.3389/feduc.2024.1364860

[30] Yang M O’Sullivan PS Irby DM Chen Z Lin C Lin C. Challenges and adaptations in implementing an English-medium medical program: a case study in China. BMC Med Educ. (2019) 19:15. doi: 10.1186/s12909-018-1452-3, PMID: 30626387 PMC6325837

[31] Kushida SS Troster EJ. Burnout prevalence in medical students attending a team-based learning school. Front Educ. (2023) 8:426. doi: 10.3389/feduc.2023.1091426, PMID: 40662108

[32] Kulas JT Stachowski AA Haynes BA. Middle response functioning in Likert-responses to personality items. J Bus Psychol. (2008) 22:251–9. doi: 10.1007/s10869-008-9064-2

[33] Nadler JT Weston R Voyles EC. Stuck in the middle: the use and interpretation of mid-points in items on questionnaires. J Gen Psychol. (2015) 142:71–89. doi: 10.1080/00221309.2014.994590, PMID: 25832738

[34] Hamad AA. Decolonization of medical education: a global screening of instructional languages and mother tongue dependence. J Med Surg Public Health. (2023) 1:100007. doi: 10.1016/j.glmedi.2023.100007

[35] Alhamami M. (2025). A burnout scale for undergraduate students in english-medium instruction. Presented at the 6th EMI symposium/3rd annual ELINET conference, transforming education, transforming lives. Department of Education, University of Oxford, UK.

